# Improvement of the glycoproteomic toolbox with the discovery of a unique C-terminal cleavage specificity of flavastacin for N-glycosylated asparagine

**DOI:** 10.1038/s41598-017-11668-1

**Published:** 2017-09-12

**Authors:** Alexander Pralow, Marcus Hoffmann, Terry Nguyen-Khuong, Erdmann Rapp, Udo Reichl

**Affiliations:** 1 Max Planck Institute for Dynamics of Complex Technical Systems, Sandtorstrasse 1, 39106 Magdeburg, Germany; 2 glyXera GmbH, Leipziger Straße 44, 39120 Magdeburg, Germany; 3 Otto-von-Guericke University, Chair of Bioprocess Engineering, Universitätsplatz 2, 39106 Magdeburg, Germany

## Abstract

To determine all potential N-glycosylation sites of a glycoprotein, one central aspect of every bottom-up N-glycoproteomic strategy is to generate suitable N-glycopeptides that can be detected and analyzed by mass spectrometry. Specific proteases, such as trypsin, bear the potential of generating N-glycopeptides that either carry more than one N-glycosylation site or are too long to be readily analyzed by mass spectrometry– both due to the lack of tryptic cleavage sites near the N-glycosylation site. Here, we present a newly identified cleavage specificity of flavastacin, a protease from *Flavobacterium menigosepticum*, which - up to now - was only reported to cleave peptide bonds N-terminal to aspartic acid residues. In contrast to literature, we could not confirm this N-terminal specificity of flavastacin for aspartic acid. However, for the first time, we show a unique cleavage specificity of flavastacin towards the C-terminus of N-glycosylated asparagine residues. Implemented in an N-glycoproteomic workflow the use of flavastacin can thus not only render data analysis much easier, it can also significantly increase the confidence of MS-based N-glycoproteomic analyses. We demonstrate this newly discovered specificity of flavastacin by in-depth LC-MS(/MS) analysis of complex-type glycosylated human lactotransferrin and bovine serum albumin peptides and N-glycopeptides that were generated by trypsin and flavastacin digestion. Following to this work, further elucidation of the efficiency, specificity and mode of action of flavastacin is needed, but we believe that our discovery has great potential to facilitate and improve the characterization of N-glycoproteomes.

## Introduction

Protein glycosylation is a co-/post-translational modification involved in several key biological functions^1^. It is becoming increasingly evident that aberrant glycosylation is associated with cancer^2^, inflammatory diseases and infectious diseases^3^. In addition, there is a rapidly expanding group of rare genetic, metabolic disorders that are due to defects in glycosylation, the so-called congenital disorders of glycosylation^4^. There are two main forms of protein glycosylation: N-linked and O-linked glycosylation. N-glycans are linked to the amino group of asparagine according to a specific consensus sequence (NXS/T; X ≠ P). Moreover, N-glycans are characterized by a common core-structure GlcNAc_2_Man_3_ (N-acetylglucosamine (GlcNAc), mannose (Man)), which can be extended to form complex-, high-mannose- or hybrid-type N-glycan structures^5^. In the past decades, the field of site-specific glycoprotein analysis methods has grown tremendously and emphasized the importance of protein glycosylation analyses with respect to micro- (different glycan structures at one glycosylation site) and macroheterogeneity (site occupancy). So far, the method of choice for site-specific glycoproteomics is the analysis of specifically or unspecifically digested glycoproteins via liquid chromatography combined with mass spectrometry (LC-MS). In addition, a glycopeptide enrichment can be performed prior to analysis, i.e. by hydrophilic interaction chromatography^6^. With regard to MS, different fragmentation strategies tackle various challenges in the field of proteomics and glycoproteomics. Lower energy collision-induced dissociation (CID) is able to generate B- and Y-glycomoiety fragment ion series of glycopeptides^7^. It allows the annotation of the glycan composition and the calculation of the peptide mass, but often lacks b- and y-peptidemoiety fragment ion series^8^ of the glycopeptides for sequence verification. Higher energy collisional dissociation (HCD) allows the adjustment of the normalized collision energy and generates peptide-specific b- and y-ions. However, due to the neutral loss of the glycan moiety, B- and Y-ion series are underrepresented^9, 10^.

**Figure 1:**
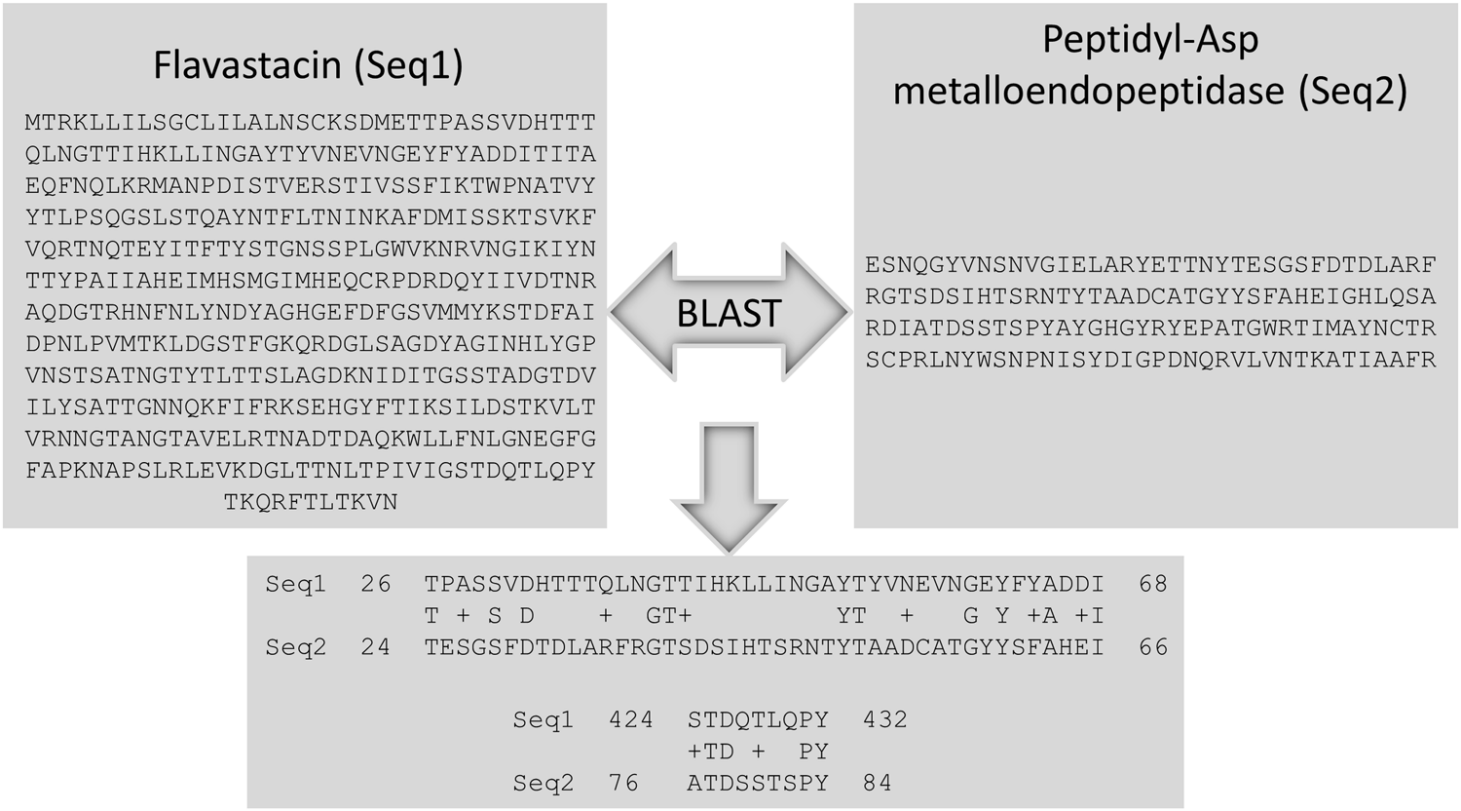
Comparison of flavastacin and peptidyl-Asp metalloendopeptidase. The protein sequences of flavastacin (Q47899) and peptidyl-Asp metalloendopeptidase (Q9R4J4) are from Uniprot. Both sequences where compared using the protein BLAST tool from the national center for biotechnology information (free online tool: https://blast.ncbi.nlm.nih.gov/Blast.cgi?-PROGRAM=blastp&PAGE_TYPE=BlastSearch&BLAST_SPEC=blast2seq&LINK_LOC=blasttab). Sequences with similarities are illustrated in the lower box. The first sequence has a similarity of 11 out of 43 amino acids (23%). The second sequence has a similarity of 4 out of 9 amino acids (44%). Possible conservative amino acid changes are indicated with “+”.

In addition to tryptic digestion of glycoproteins, further sequential treatment with other proteases is used to overcome low charge density and sequence constraints of glycopeptides with a large peptide moiety. The endoproteinase AspN is a zinc metalloendoproteinase produced in *Pseudomonas fragi* (Boehringer Ingelheim, Uniprot: Q9R4J4), which selectively cleaves peptide bonds N-terminal to aspartic acid^11^. AspN is also known for N-terminal cleavage at cysteine and glutamic acid^12, 13^. Its primary use in glycoproteomic experiments so far, has involved the cleavage of deamidated asparagine after N-glycan release by peptide N-glycosidase F (PNGaseF) to assess N-glycan presence and location^14^. Flavastacin (New England Biolabs, Uniprot: Q47899), which is produced in *Flavobacterium menigosepticum*, has been described to behave similar to the AspN from *Pseudomonas fragi* ^15, 16^. Therefore, is also called AspN, despite its quite different amino acid sequence and thus, protein identity. However, both proteins belong to the family of metalloendoproteases. A BLAST search comparing these two proteins shows no overlapping sequences and only two short segments with quite low similarity (see Fig. 1). Thus, the two sequences cannot be aligned, which renders common protein functions unlikely.

This work describes a new and unique protease specificity of flavastacin for the C-terminus of N-glycosylated asparagine. In contrast to literature^15, 16^, no specificity for the N-terminus of aspartic acid was observed. Subsequent analysis of this unexpected phenomenon via *de-novo* sequencing of the resulting N-glycopeptide fragment ion spectra led to the discovery of this previously unknown cleavage specificity of flavastacin. Our findings provide investigators with a new tool for targeted N-glycoprotein digestion to overcome common problems in N-glycoproteomics, like large N-glycopeptides with too many amino acids for proper LC-MS measurements, or multiple N-glycosylation sites within one N-glycopeptide.

## Materials and Methods

### Chemicals

The proteins bovine serum albumin (BSA; A3912–100G) and lactotransferrin from human milk (hLTF; L4894-5MG) were purchased from Sigma-Aldrich. Enzymes used for digestion were trypsin (Trypsin Sequencing Grade Modified; V5111) from Promega and endoproteinase AspN (AspN; P8104S) from New England Biolabs (Flavastacin, purified host cell protein, Uniprot Q47899; see the corresponding SDS-gel for purity of AspN (Flavastacin) in Supplementary Figure 1). All solvents for LC were MS grade. All buffer and solutions were prepared with deionized and purified water (dH_2_O) using a Milli-Q water purification system (18.2 MΩ · cm^−1^ at 25°C, total organic carbon of 3 ppb) from Merck Millipore. For LC-MS solvents, water was further purified using the LC-Pak Polisher from Merck Millipore.

### Proteolytic Digestion via Filter-Aided Sample Preparation (FASP)

BSA and hLTF were digested proteolytically using a modified version of the filter-aided sample preparation (FASP) method^17^. Briefly, 100 µg of each protein were applied to a filter unit (Nanosep® Omega™ with polyethersulfone membrane, molecular weight cut-off 10 kDa; PALL Life Sciences). Samples were treated with urea buffer_(Tris-HCl)_ (8 M urea in 0.1 M Tris-HCl_(aq)_ pH 8.5; AppliChem), followed by reduction with DL-dithiothreitol (40 mM DTT, Sigma-Aldrich), and alkylation with iodoacetamide (55 mM IAA, Sigma-Aldrich) – each dissolved in 50 mM ammonium bicarbonate_(aq)_ (ABC buffer_(aq)_, Sigma-Aldrich). Each filter unit was washed three times with urea buffer_(Tris-HCl)_ and three times with ABC buffer_(aq)_. Proteins were digested proteolytically with trypsin using an enzyme/protein ratio of 1:30 (w/w). Samples were incubated overnight at 37°C and 350 rpm using a temperature controlled incubator (Titramax 1000 + Inkubator 1000, Heidolph). Digests were collected by centrifugation. Filter units were washed twice, first using 50 µL ABC buffer_(aq)_ with 5%_(v/v)_ acetonitrile (ACN), then using 50 µL dH_2_O; in between samples were centrifuged. The flow through was kept along with the digest, in order to be dried by vacuum centrifugation.

After tryptic digestion, approximately 20 µg peptides were reconstituted in 20 µl 1x AspN reaction buffer (New England Biolabs; 50 mM Tris-HCL, 2.5 mM Zinc Sulfate, pH 8.0). Afterwards AspN (Flavastacin) was added (enzyme/protein ratio 1:20) to the peptide solution and incubated overnight at 37°C as recommended by the supplier. The enzyme reaction was stopped via centrifugation trough a filter unit (same as described above). The flow through (tryptic digests of BSA and hLTF, as well as the sequential digests of BSA and hLTF with trypsin and AspN (Flavastacin) was dried by vacuum centrifugation and reconstituted in 0.1%_(v/v)_ trifluoroacetic acid_(aq)_ (TFA; Thermo Fisher Scientific) prior to LC-MS(/MS) measurements.

### LC-MS(/MS) Measurement

The LC system was an UltiMate 3000 Rapid Separation LC system from Thermo Fisher Scientific. Samples (≈500 ng) were loaded isocratically on a trap column (Acclaim PepMap®100, 100 µm × 2 cm nanoViper C18, 5 µm, 100 Å, Thermo Fisher Scientific) via 100% loading buffer A (98%_(v/v)_ dH_2_O, 2%_(v/v)_ ACN, 0.05%_(v/v)_ TFA) with a flow rate of 7 µL/min within the first 5 min. Afterwards, the loaded trap column was switched in line with the separation column (Acclaim PepMap®RSLC, 75 µm × 25 cm nanoViper C18, 2 µm, 100 Å, Thermo Fisher Scientific), with a nano flow rate of 0.3 µL/min of 4% nano buffer B (10%_(v/v)_ dH_2_O, 10%_(v/v)_ trifluoroethanol (TFE), 80%_(v/v)_ ACN, 0.1%_(v/v)_ formic acid (FA)) and nano buffer A (98%_(v/v)_ dH_2_O, 2%_(v/v)_ ACN, 0.1%_(v/v)_ FA). The separation was performed by a multi-step binary nano A/B gradient: 4–55% nano buffer B till 80 min, 55–90% nano buffer B till 100 min, 90% nano buffer B till 110 min and 4% nano buffer B 110–150 min.

The eluting peptides were measured on an LTQ Orbitrap Elite mass spectrometer from Thermo Fisher Scientific using a Nanospray Flex^TM^ source in positive ionization mode with a capillary voltage of −2.7 kV. Peptides and glycopeptides were fragmented using HCD with normalized collision energy of 35 with an activation time of 0.1 ms. The five most intense precursor ions with a charge state >1 were chosen for fragmentation. The recorded mass range for MS was 350–2000 m/z and for MS/MS 150–2000 m/z.

### Data Analysis

The complete MS(/MS) data for hLTF (Trypsin + AspN (Flavastacin)) were analyzed manually using *Xcalibur* (Version 2.2, Qual Browser, Thermo Fisher Scientific). The first step was the recognition of glycopeptide related fragment ion spectra due specific B-ions “oxonium ions”. Afterwards, the peptide mass was presumed by a specific fragmentation pattern: [peptide – NH_3_ + H^+^]; [peptide + H^+^] and [peptide + GlcNAc + H^+^]. Using *ExPASy Findpept* (free online tool: web.expasy.org/findpept) the presumed peptide mass was screened against an unspecific digestion of hLTF (Uniprot; P02788) with a maximum mass tolerance of 0.02 Da, carbamidomethylation of cysteine and the oxidation of methionine. The presumed peptide sequence was furthermore *in-silico* fragmented using *MS-Product* (free online tool: prospector.ucsf.edu/prospector/cgi-bin/msform.cgi?form = msproduct). The b- and y-ions were compared with the corresponding ions in the MS/MS fragment ion spectrum with a maximum mass tolerance of 0.02 Da to validate the presumed peptide sequence. The mass difference between the peptide mass and the precursor mass was used to predict the N-glycan composition using *ExPASy GlycoMod* (free online tool: web.expasy.org/glycomod).

The MS(/MS) data from BSA and hLTF were imported into *Proteome Discoverer* (Version 1.4, Thermo Fisher Scientific) and searched against UniProt-KB/SwissProt database (542258 sequences; downloaded January, 2014) using *MASCOT* (Version 2.5, Matrix Science). The MS(/MS) data were screened against an unspecific *in-silico* digestion of the mammalian taxonomy database with the fixed modification of cysteine with carbamidomethyl, variable deamidation of asparagine, and variable oxidation of methionine. The precursor ion mass tolerance was set to 5 ppm and the fragment ion mass tolerance to 0.02 Da. The protein relevance threshold was set to 20 and the peptide cut off score to 10. The target false discovery rate for peptide hits was set to 0.01 (strict setting, and to 0.05 as relaxed setting).

### Data availability statement

The datasets generated during and/or analyzed during the current study are available from the corresponding author on reasonable request.

## Results and Discussion

Every N-glycoproteomic analysis workflow consists of numerous parameters to be optimally adjusted. In particular, the design of proteolytic digestion using sequential digestion steps with a selection of specific enzymes is an important step to overcome common problems such as too large N-glycopeptides (with low charge density and/or sequence constraints) and N-glycopeptides with multiple glycosylation sites. Here, we present a new approach for the proteolytic digest of glycoproteins by using flavastacin, a protease that we found to cleave specifically at the C-terminus of N-glycosylated asparagine. The glycoprotein hLTF and the non-glycosylated protein BSA were used as model proteins to demonstrate this newly identified cleavage specificity. According to the manufacturer’s recommendation, flavastacin works only on peptides smaller than 50 amino acids. Therefore, hLTF and BSA were first treated with trypsin before flavastacin was added (see Materials and Methods).

The hLTF is a well-characterized glycoprotein present in human milk, saliva, tears, nasal secretions and other body fluids^18^ that contains three potential N-glycosylation sites (N_156_, N_497_, and N_642_). The sites N_156_ and N_497_ are described to carry complex-type mostly core-fucosylated and sialylated N-glycans, also including N-acetyllactosamine (LacNAc) extensions^19^. For site N_642_ no N-glycosylation has been identified so far. The theoretical tryptic N-glycopeptides of hLTF are (R)PFL**N**_**156**_WTGPPEPIEAAVAR(F) (1964.0155 Da) (with a commonly missed cleavage due to proline: (R)TAGWNVPIGTLRPFLN_156_WTGPPEPIEAAVAR(F) (3229.7036 Da)), (R)TAGWNIPMGLLF**N**_**497**_QTGSCK(F) (2036.9812 Da) and (R)**N**_**642**_GSDCPDK(F) (834.3178 Da). These peptides consist of 18/(30), 19 and 8 amino acids, respectively. The large peptide moieties of the sites N_156_ and N_497_ in combination with an N-glycosylation can reach masses, which are unsuitable for proper N-glycoproteomic analysis^20^.

**Figure 2:**
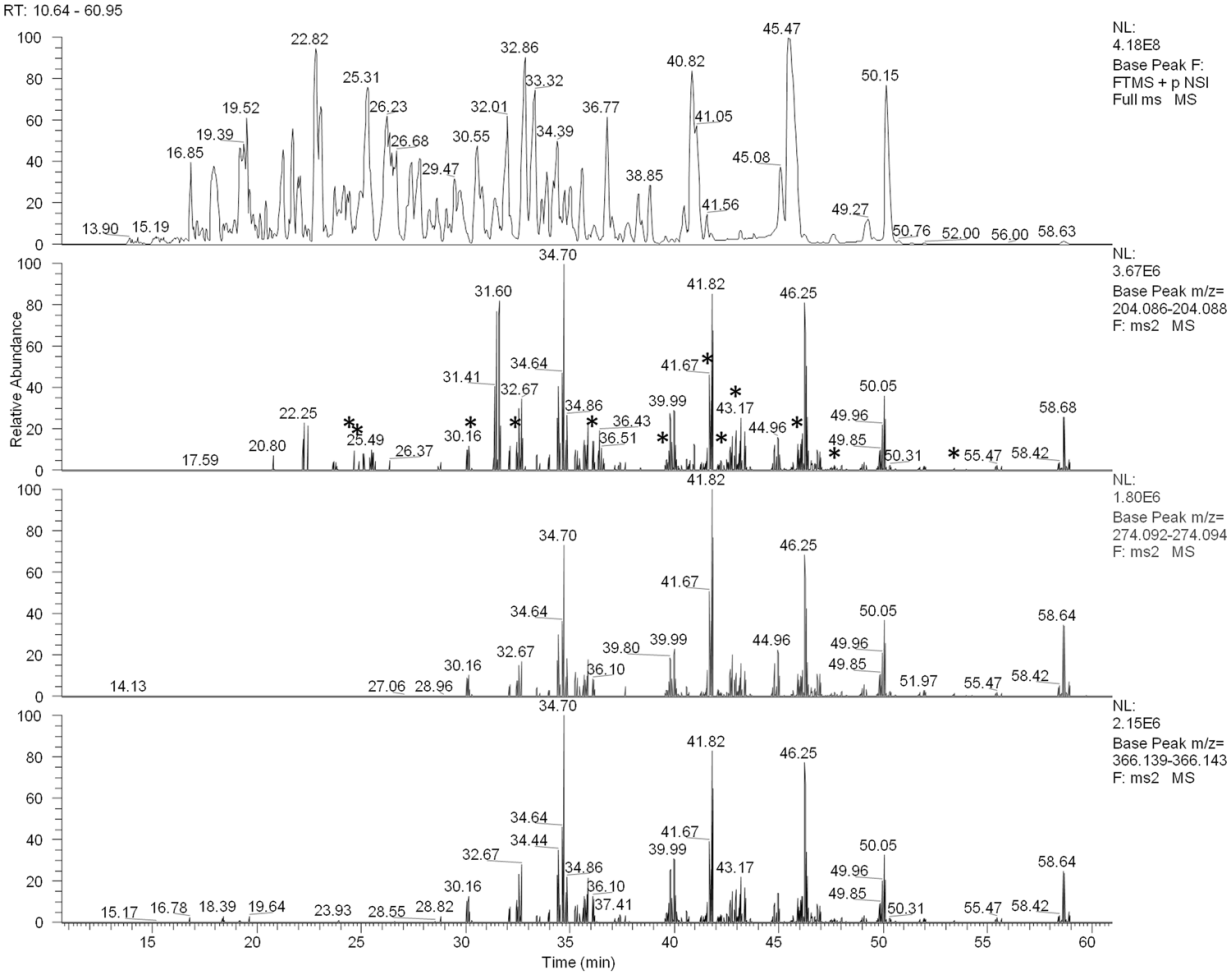
Base peak ion-chromatogram (BPC) and oxonium ion related extracted ion-chromatograms (EIC) of MS(/MS) spectra of nanoRP-LC-ESI-OT-MS^2^ (HCD) measured hLTF after sequential digestion with trypsin and flavastacin. Starting from the top: BPC of MS spectra (grey), EIC of MS/MS spectra of HexNAc within the m/z range 204.086–204.088 [M + H]^+^ (red), EIC of MS/MS spectra of NeuAc-H_2_O within the m/z range 274.092–274.094 (green), EIC of MS/MS spectra of HexHexNAc within the m/z range 366.139–366.143 (blue). The ion-chromatograms are illustrated in the time range 10.64–60.95 min. The accepted mass error of the EIC of the specific oxonium ions is 5 ppm. Signals listed in Table 1 are marked using asterisks.

N-glycoproteomic analysis of flavastacin-generated hLTF N-glycopeptides revealed that all detected N-glycopeptides feature the N-glycosylated asparagine at the C-terminus, while the N-terminus was either a tryptic or an unspecific cleavage site. In addition, the generated N-glycopeptides were shorter compared to a solely tryptic digest. The identified N-glycopeptides for the N-glycosylation sites N_156_ and N_497_ are 4–16, and 4–13 amino acids long, respectively (see Table 1). In agreement with literature, no N-glycopeptide was detected for the N-glycosylation site N_642_^19^. We did not perform any further enrichment of glycopeptides prior to MS analysis since the digestion strategy together with a long separation gradient and a high-resolution mass spectrometry measurement resulted in a comprehensive coverage of glycopeptides as shown in Fig. 2 (for solely tryptic digest see Supplementary Figure 2). The eluting N-glycopeptides are depicted by extracted ion chromatograms of MS/MS spectra containing glycan-specific oxonium ions: *m/z* 204.087 (N-acetylhexosamine (HexNAc) [M + H]^+^), *m/z* 274.093 (N-acetylneuraminic acid (NeuAc)-H_2_O [M + H]^+^) and *m/z* 366.141 (hexose (Hex) + HexNAc [M + H]^+^). The unspecific cleavage specificity due to flavastacin results in redundant N-glycopeptide signals over the elution time range with peptide moieties of different length. This can be a disadvantage for the analysis of more complex glycopeptide samples.

**Table 1:** Manually annotated N-glycopeptide sequences of human lactotransferrin (hLTF) from MS(/MS) spectra of nanoRP-LC-ESI-OT-MS^2^ (HCD) measurements after sequential digestion of hLTF with trypsin and flavastacin.

N-Glycosylation Site	Experimental/Observed mass [Da]	Theoretical mass [Da]	Δ Mass [Da]	Peptide	Position	Modifications	RT [min]
156	490.2640	490.2660	0.002	(R)PFLN(W)	153–156		24.73
	860.4960	860.4990	0.003	(G)TLRPFLN(W)	150–156		25.07
	1127.6620	1127.6570	−0.005	(V)PIGTLRPFLN(W)	147–156		40.09
	1226.7230	1226.7260	0.003	(N)VPIGTLRPFLN(W)	146–156		41.54
	1755.9520	1755.9540	0.002	(R)TAGWNVPIGTLRPFLN(W)	141–156		42.17
	1340.7680	1340.7680	0.000	(W)NVPIGTLRPFLN(W)	145–156		42.93
497	856.4950	856.4961	−0.001	(N)IPM(−48)GLLFN(Q)	490–497	M(CAM) (−105)	30.11
	506.2990	506.2970	−0.002	(G)LLFN(Q)	494–497		32.10
	563.3210	563.3190	−0.002	(M)GLLFN(Q)	493–497		36.07
	1385.7223	1385.7246	−0.002	(R)TAGWNIPM(−48)GLLFN(Q)	485–497	M(CAM) (−105)	39.58
	920.4930	920.4910	−0.002	(N)IPMGLLFN(Q)	490–497	MSO	39.95
	904.4950	904.4960	0.001	(N)IPMGLLFN(Q)	490–497		45.56
	791.4110	791.4120	0.001	(I)PMGLLFN(Q)	491–497		47.45
	694.3590	694.3590	0.000	(P)MGLLFN(Q)	492–497		53.33

CAM - carbamidomethylation; MSO - methionine S-oxidation.

**Figure 3:**
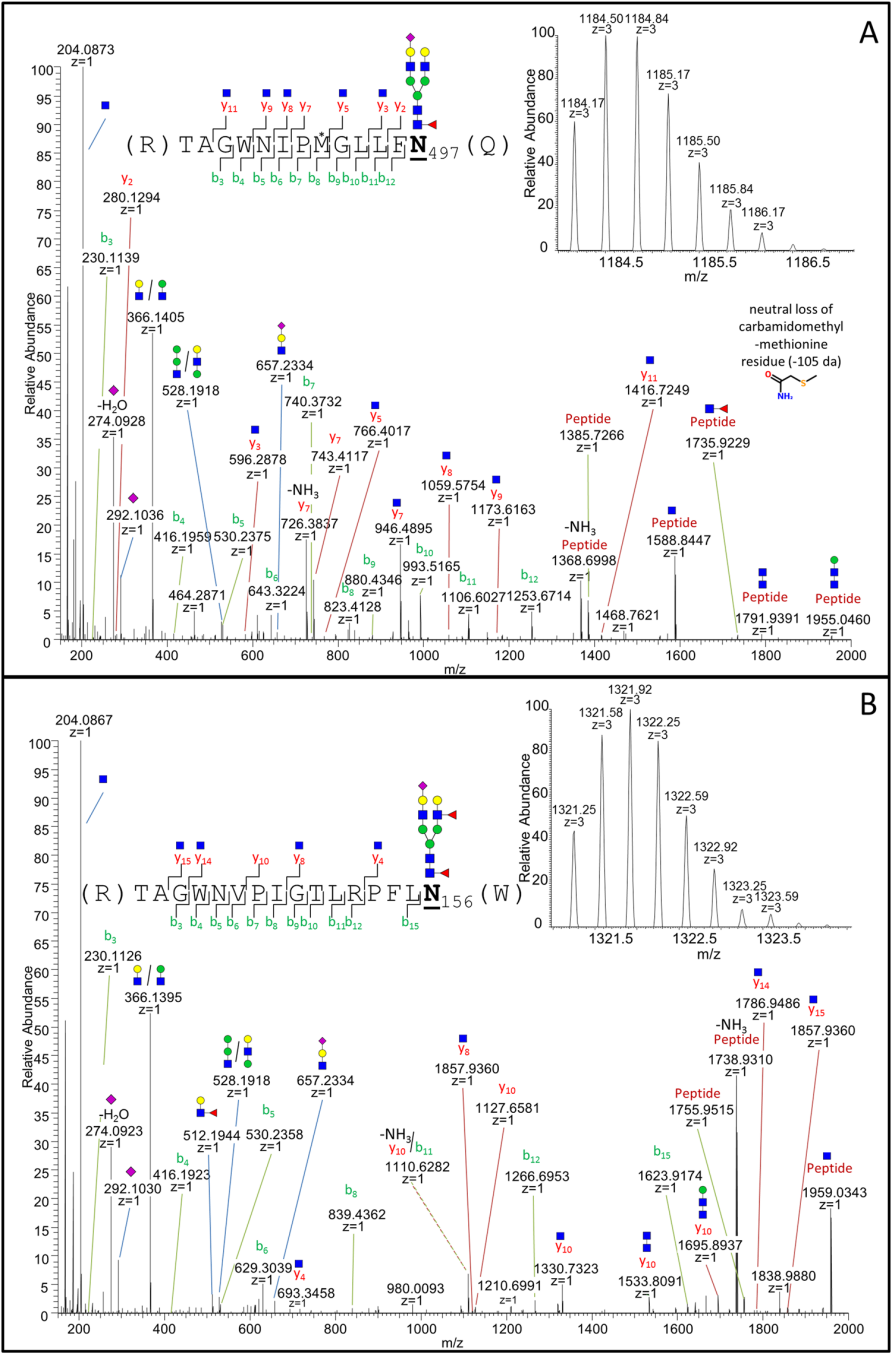
Fragment ion spectra of nanoRP-LC-ESI-OT-MS^2^ (HCD) measured hLTF N-glycopeptides after sequential digestion with trypsin and flavastacin. (**A**) For site N_497_ the fragment ion spectrum of the N-glycopeptide sequence TAGWNIPM*GLLFN_497_ with the N-linked glycan Hex_5_HexNAc_4_dHex_1_NeuAc_1_ and the corresponding precursor ion m/z 1184.1659 [M + 3 H]^3+^ is shown. The “*” indicates the carbamidomethylation of methionine. (**B**) For site N_156_ the fragment ion spectrum of the N-glycopeptide sequence TAGWNVPIGTLRPFLN_156_ with the N-linked glycan Hex_5_HexNAc_4_dHex_2_NeuAc_1_ and the corresponding precursor ion m/z 1321.2479 [M + 3 H]^3+^ is shown. N-glycan structures and oxonium ions are illustrated according to CFG nomenclature^22^. The b-ion series are highlighted in green, the y-ion series in red and the B-ions in blue. The isotopic patterns of the precursors are shown at the upper right corner.

To illustrate the unique cleavage specificity of flavastacin, two examples for annotated MS/MS spectra of the N-glycopeptide containing sites N_497_ (Fig. 3A) and N_156_ (Fig. 3B) are shown. Both N-glycopeptides have the N-glycosylated asparagine at the C-terminus and the tryptic cleavage site at the N-terminus. For the site N_497_, the fragment ion spectrum of the N-glycopeptide TAGWNIPM*GLLF**N**_**497**_ with the N-linked glycan Hex_5_HexNAc_4_dHex_1_NeuAc_1_ (deoxyhexose (dHex)) (precursor ion: m/z 1184.1659 [M + 3 H]^3+^) is depicted, along with the corresponding peptide ion 1385.7266 [M + H]^+^ (see Fig. 3A). The example for the fragment ion spectrum of site N_497_ shows a neutral loss of 105 Da, related to a carbamidomethyl-methionine residue (see Fig. 3A). This neutral loss from a carbamidomethylated methionine has been described only rarely in literature^21^. Without the awareness of a carbamidomethylation of methionine, only a neutral loss of 48 Da would be recognized, which can mistakenly also be interpreted as a side chain loss of methionine sulfoxide. However, due to the specific digestion strategy involving both trypsin and flavastacin, in combination with high-resolution LC-MS, the identification of such unlikely modifications is also possible. For site N_156_, the fragment ion spectrum of the N-glycopeptide TAGWNVPIGTLRPFL**N**_**156**_ with the N-linked glycan Hex_5_HexNAc_4_dHex_2_NeuAc_1_ (precursor ion: m/z 1321.2479 [M + 3 H]^3+^) is depicted (see Fig. 3B) as well as the corresponding peptide ion 1755.9515 [M + H]^+^. Both fragment ion spectra are dominated by the b-ion series, oxonium ions (B-ions) and the aforementioned fragmentation pattern: [peptide - NH_3_ + H^+^]; [peptide + H^+^], and [peptide + GlcNAc + H^+^] (Fig. 3). MS/MS spectra of additional N-terminal unspecifically cleaved N-glycopeptides are shown in Supplementary Figures 3 and 4).

Analysis of flavastacin-generated BSA peptides revealed primarily tryptic cleavage at the C-terminus (34 of 37 peptides), and tryptic and unspecific cleavage at the N-terminus (13 tryptic, 24 unspecific cleavages). Twelve peptides had tryptic cleavages at both termini (see Table 2). For the non-glycosylated BSA no flavastacin-generated peptides were detected with an asparagine at the C-terminus, which correlates to observations we made for non-glycosylated asparagines of the N-glycosylated hLTF (see Supplementary Table 1). To check for possible unspecific cleavages of the tryptic digest and its influence on the flavastacin digest, identified peptides of solely tryptically digested BSA (see Supplementary Table 2) and hLTF (see Supplementary Table 3) were examined. Here, almost exclusively, specific cleavages were identified in the tryptic digests of BSA and hLTF. This strongly suggests that the observed N-glyco-specific cleavage of hLTF, as well as the unspecific cleavage of BSA and hLTF of the combined digest (trypsin and flavastacin) can only be linked to the activity of flavastacin.

**Table 2:** Proteome Discoverer results of a nanoRP-LC-ESI-OT-MS^2^ (HCD) measurement after sequential digestion of BSA with trypsin and flavastacin.

Sequence	# PSM	Modifications	MH + [Da]	IonScore	ΔM [Da]
(K)TVMENFVAFVDK(C)	2	N5(Deamidated)	1400.67510	107	−0.002
(R)MPCTEDYLSLILNR(L)	2	C3(Carbamidomethyl)	1724.82927	98	−0.005
(K)LGEYGFQNALIVR(Y)	42	N8(Deamidated)	1480.77873	98	0.004
(K)LGEYGFQNALIVR(Y)	13		1479.79204	97	0.000
(K)TVMENFVAFVDK(C)	9		1399.69121	93	−0.001
(K)TVMENFVAFVDK(C)	1	M3(Oxidation)	1415.68498	93	−0.003
(K)VPQVSTPTLVEVSR(S)	4		1511.84062	86	−0.002
(P)CTEDYLSLILNR(L)	3	C1(Carbamidomethyl)	1496.73870	83	−0.002
(R)RHPEYAVSVLLR(L)	6		1439.81028	82	−0.002
(M)ENFVAFVDK(C)	7		1068.53703	77	0.001
(R)KVPQVSTPTLVEVSR(S)	2		1639.93723	76	−0.001
(K)DAFLGSFLYEYSR(R)	2		1567.73613	76	−0.007
(E)YGFQNALIVR(Y)	6	N5(Deamidated)	1181.63017	75	−0.001
(G)EYGFQNALIVR(Y)	5	N6(Deamidated)	1310.67229	74	0.000
(M)ENFVAFVDK(C)	2	N2(Deamidated)	1069.51872	71	−0.001
(K)LGEYGFQNAL(I)	2		1111.54180	68	0.000
(K)KQTALVELLK(H)	13		1142.71416	68	−0.000
(Y)FYAPELLYYANK(Y)	1		1491.75029	68	−0.002
(K)LVNELTEFAK(T)	1	N3(Deamidated)	1164.61382	65	−0.001
**(E)DYLSLILNR(L)**	4		1106.62212	63	0.002
(K)DAIPENLPPLTADFAEDK(D)	2		1955.95671	60	−0.003
(S)TPTLVEVSR(S)	1		1001.56133	57	−0.001
(K)HLVDEPQNLIK(Q)	2		1305.71526	57	−0.001
(R)HPEYAVSVLLR(L)	2		1283.70691	56	−0.004
(P)EYAVSVLLR(L)	3		1049.59648	56	−0.003
(E)YAPELLYYANK(Y)	1		1344.68120	55	−0.002
(Q)VSTPTLVEVSR(S)	1		1187.66106	55	−0.002
(F)LGSFLYEYSR(R)	3		1234.60918	55	−0.001
(N)FVAFVDK(C)	2		825.44951	55	−0.001
(N)LPPLTADFA(E)	7		944.50633	54	−0.001
**(A)IPENLPPLTA(D)**	5		1064.59661	54	−0.002
(Y)APELLYYANK(Y)	1		1181.61870	54	−0.001
(T)ALVELLK(H)	1		785.51219	54	−0.001
(E)YAVSVLLR(L)	2		920.55394	52	−0.002
(K)LVNELTEFAK(T)	1		1163.62810	52	−0.003
(Q)TALVELLK(H)	3		886.55974	48	−0.001
(G)FQNALIVR(Y)	1		960.56017	47	−0.002

MASCOT search against unspecific *in-*silico digestion of mammalian taxonomy (UniProt-KB/SwissProt database). Peptide sequences with the N-terminal cleavage of aspartic acid or deamidated asparagine (as indicated from the supplier of flavastacin) are underlined and bold.

Whilst it has been described that flavastacin has specificities towards the N-terminus of aspartic acid, glutamic acid and cysteine, we found a unique cleavage specificity of flavastacin for the C-terminus of N-glycosylated asparagine, which was not explored up to now. All manually annotated hLTF N-glycopeptide related peptide sequences are listed in Table 1 (other identified non-glycosylated peptides are listed in Supplementary Table 1). Every single hLTF N-glycopeptide sequence has been cleaved at the N-glycosylated asparagine at the C-terminus – independent of the N-glycoform attached to the respective N-glycosylation site. In addition, we observed that the N-terminus is a tryptic or an unspecific cleavage site. Based on the manually annotated peptide sequences and the database-assisted MASCOT search, no strict N-terminal cleavage of aspartic acid could be observed, neither for BSA nor for hLTF (Tables 1 and 2, Supplementary Table 1).

## Conclusion and Outlook

**Figure 4:**
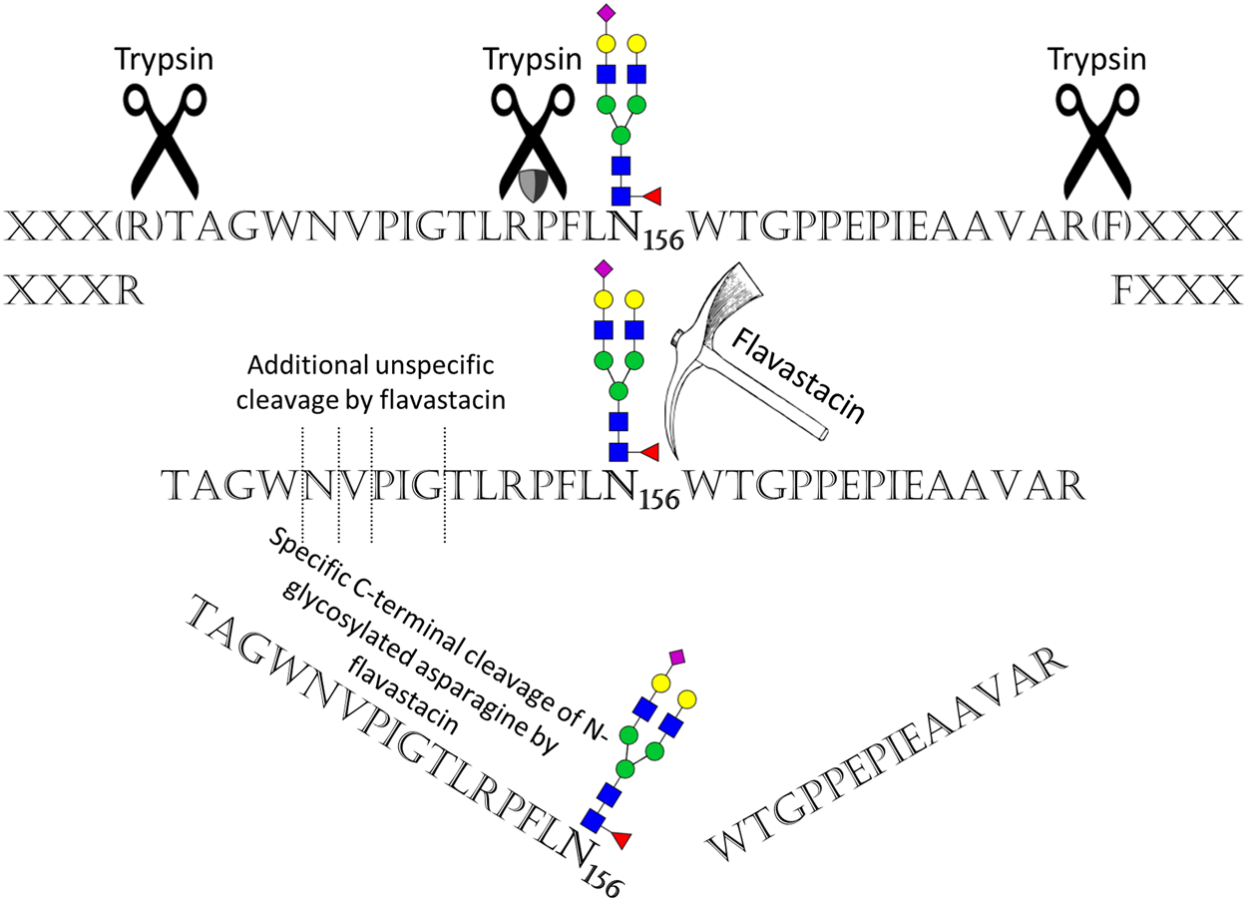
Specificity of flavastacin for the C-terminus of N-glycosylated asparagine. The amino acid sequence of the tryptic N-glycopeptide of hLTF with the N-glycan Hex_5_HexNAc_4_dHex_1_NeuAc_1_ linked to N-glycosylation site N156 is shown. The scissors symbolize trypsin; the shield stands for cleavage inhibition of trypsin due to proline (P). Flavastacin is symbolized by a pick, and its specific C-terminal cleavage of N-glycosylated asparagine, as well as its unspecific N-terminal cleavages are shown. The N-glycan structure is illustrated according to CFG nomenclature^22^.

Flavastacin shows a clear specificity for the C-terminus of N-glycosylated asparagine N_156_ and N_497_ in hLTF (illustrated in Fig. 4). Due to the presence of multiple N-glycosylation sites and the well-described complex-type N-glycan structures, hLTF is a very suitable glycoprotein to demonstrate this newly found specificity of flavastacin.

In contrast to previous work^15, 16^, we could not verify the claimed specificity of flavastacin for the N-terminus of aspartic acid, neither for hLTF nor for BSA. However, we could demonstrate that the sequential combination of trypsin and flavastacin for protein digestion successfully cleaves the N-glycoprotein hLTF in well annotatable N-glycopeptide sequences. Interestingly, in contrast to unspecific digestion strategies using proteinase K or pronase, flavastacin works as an “N-glyco-specific” proteolytic enzyme (specific for N-glycosylated asparagine at the C-terminus). This property improves data quality as well as data analysis and therefore facilitates N-glycoproteomics significantly. However, the unspecific cleavage due to flavastacin at the N-terminus results in the distribution of redundant N-glycopeptide signals with peptide moieties of different length.

Overall, this finding improves the glycoproteomic toolbox and helps to overcome common problems in N-glycoproteomics, i.e. the presence of too large N-glycopeptides with too many amino acids and/or too many N-glycosylation sites for proper LC-MS analysis. Despite the fact that this specificity of flavastacin and its cleaving mechanism need to be examined also for other glycoproteins (as well as for more complex (glyco-)protein mixtures), and in particular for other types of glycosylation (like high-mannose-type, hybrid-type and O-glycosylation), the use of flavastacin will already be beneficial for glycoscience now, as it allows researchers to dig faster and deeper into N-glycoproteomes.

## Electronic supplementary material

Supplementary Information
