# Supplementary material for: Improvement of the glycoproteomic toolbox with the discovery of a unique C-terminal cleavage specificity of flavastacin for N-glycosylated asparagine
Source: Sci Rep. 2017 Sep 12;7:11419. doi: 10.1038/s41598-017-11668-1 (PMC5595805; doi:10.1038/s41598-017-11668-1)
Supplement: Supplementary file 1 — Supplementary Information [file 41598_2017_11668_MOESM1_ESM.docx]

Improvement of the glycoproteomic toolbox with the discovery of a unique C-terminal cleavage specificity of flavastacin for N-glycosylated asparagine

Alexander Pralow ^1^, Marcus Hoffmann ^1^, Terry Nguyen-Khuong ^1^, Erdmann Rapp ^1,2^, Udo Reichl ^1,3^

^1^ Max Planck Institute for Dynamics of Complex Technical Systems, Sandtorstrasse 1, 39106 Magdeburg, Germany

^2^ glyXera GmbH, Leipziger Straße 44, 39120 Magdeburg, Germany

^3^ Otto-von-Guericke University, Chair of Bioprocess Engineering, Universitätsplatz 2, 39106 Magdeburg, Germany

Corresponding Author: Dr. rer. nat. Erdmann Rapp (rapp@mpi-magdeburg.mpg.de)

**Supplementary**

Supplementary Figure 1: SDS-Gel of flavastacin with coomassie-blue staining. 10 µg of flavastacin were loaded on a 12% bis-tris gel using MOPS running buffer. The marker is the PageRuler™ Plus Prestained Protein Ladder.

Supplementary Figure 2: Base peak ion-chromatogram (BPC) and oxonium ion related extracted ion-chromatograms (EIC) of MS(/MS) spectra of nanoRP-LC-ESI-OT-MS^2^(HCD) measured hLTF after digestion with trypsin. Starting from the top: BPC of MS spectra, EIC of MS/MS spectra of HexNAc within the m/z range 204.086-204.088 [M+H]^+^, EIC of MS/MS spectra of NeuAc-H_2_O within the m/z range 274.092-274.094, EIC of MS/MS spectra of HexHexNAc within the m/z range 366.139-366.143. The ion-chromatograms are illustrated in the time range 9.97-63.50 min. The accepted mass error of the EIC of the specific oxonium ions is 5 ppm.

Supplementary Figure 3: MS^2^ spectrum of a N-terminal unspecific cleaved N-glycopeptide of the site N156. Fragment ion spectrum of nanoRP-LC-ESI-OT-MS^2^(HCD) measured hLTF N-glycopeptides after sequential digestion with trypsin and flavastacin.

Supplementary Figure 4: MS^2^ spectrum of an N-terminal unspecific cleaved N-glycopeptide of the site N497. Fragment ion spectrum of nanoRP-LC-ESI-OT-MS^2^(HCD) measured hLTF N-glycopeptides after sequential digestion with trypsin and flavastacin.

Supplementary Table 1: Proteome Discoverer results of a nanoRP-LC-ESI-OT-MS^2^(HCD) measurement after sequential digestion of hLTF with trypsin and flavastacin. MASCOT search against unspecific *in-*silico digestion of mammalian taxonomy (UniProt-KB/SwissProt database). Peptide sequences with the N-terminal cleavage of aspartic acid or deamidated asparagine (as indicated from the supplier of flavastacin) are highlighted in red.

| **Sequence** | **# PSM** | **Modifications** | **MH+ [Da]** | **IonScore** | **ΔM [Da]** |
| --- | --- | --- | --- | --- | --- |
| AVTLDGGFIYEAGLAPYK | 2 |  | 1884.96916 | 118 | -0.005 |
| VTLDGGFIYEAGLAPYK | 2 |  | 1813.93694 | 115 | 0.000 |
| ADAVTLDGGFIYEAGLAPYK | 8 |  | 2071.03203 | 115 | -0.005 |
| DVTVLQNTDGNNNEAWAK | 3 |  | 1988.93157 | 106 | 0.001 |
| GEADAMSLDGGYVYTAGK | 3 |  | 1804.80339 | 105 | -0.002 |
| DAMSLDGGYVYTAGK | 3 |  | 1547.70452 | 96 | 0.000 |
| PFLNWTGPPEPIEAA | 3 | N4(Deamidated) | 1639.79436 | 94 | -0.006 |
| MSLDGGYVYTAGK | 2 |  | 1361.63091 | 92 | -0.009 |
| LNWTGPPEPIEAAVAR | 3 | N2(Deamidated) | 1721.88347 | 85 | -0.002 |
| AMSLDGGYVYTAGK | 4 |  | 1432.66716 | 85 | -0.011 |
| SLDGGYVYTAGK | 6 |  | 1230.59307 | 82 | -0.007 |
| KGGSFQLNELQGLK | 8 |  | 1518.81743 | 82 | -0.010 |
| GGSFQLNELQGLK | 7 |  | 1390.73186 | 81 | -0.001 |
| SDTSLTWNSVK | 4 | N8(Deamidated) | 1238.58220 | 81 | -0.008 |
| KGGSFQLNELQGLK | 5 | N8(Deamidated) | 1519.80046 | 78 | -0.011 |
| QLFGSPSGQK | 2 |  | 1048.54045 | 77 | -0.002 |
| DGAGDVAFIR | 6 |  | 1020.50469 | 77 | -0.006 |
| PVAAEVYGTER | 1 |  | 1191.59941 | 76 | -0.001 |
| DGGFIYEAGLAPYK | 4 |  | 1500.73662 | 73 | 0.000 |
| GGSFQLNELQGLK | 2 | N7(Deamidated) | 1391.71404 | 73 | -0.003 |
| (L)DGGYVYTAGK(C) | 4 |  | 1030.48296 | 72 | -0.001 |
| IYEAGLAPYK | 2 |  | 1124.59172 | 70 | -0.007 |
| LRPVAAEVYG | 1 |  | 1074.58684 | 70 | -0.007 |
| LDGGYVYTAGK | 4 |  | 1143.56816 | 69 | -0.002 |
| TAGWNVPIGTLRPF | 3 |  | 1528.82671 | 67 | 0.000 |
| (L)NWTGPPEPIEAAVAR(F) | 3 | N1(Deamidated) | 1608.80254 | 67 | 0.001 |
| THYYAVAVVK | 2 |  | 1150.62476 | 63 | -0.001 |
| RSDTSLTWNSVK | 2 |  | 1393.70500 | 63 | -0.002 |
| LRPVAAEVYGTER | 4 |  | 1460.78345 | 63 | -0.002 |
| (L)DGGFIYEAGL(A) | 1 |  | 1041.48467 | 63 | -0.004 |
| (L)DGGFIYEAGLAPY(K) | 1 |  | 1372.63616 | 62 | -0.006 |
| TAGWNVPIGTLR | 8 |  | 1284.70586 | 61 | 0.000 |
| LNWTGPPEPIEAAVAR | 2 |  | 1720.90019 | 60 | -0.002 |
| LRPVAAEVY | 1 |  | 1017.56499 | 60 | -0.008 |
| VPIGTLRPF | 2 |  | 999.59953 | 59 | 0.001 |
| EPIEAAVAR | 4 |  | 955.51976 | 59 | -0.001 |
| WTGPPEPIEAAVAR | 1 |  | 1493.77837 | 59 | 0.004 |
| YYGYTGAFR | 9 |  | 1097.49968 | 59 | -0.005 |
| SDTSLTWNSVK | 2 |  | 1237.59917 | 58 | -0.007 |
| EDAIWNLLR | 3 | N6(Deamidated) | 1130.58367 | 57 | 0.000 |
| TAGWNVPIGTLRP | 1 |  | 1381.75627 | 57 | -0.002 |
| GPQYVAGITNLK | 1 |  | 1260.68547 | 57 | -0.009 |
| TAIQNLR | 1 |  | 815.47423 | 56 | 0.001 |
| VAGITNLK | 1 | N6(Deamidated) | 816.48180 | 54 | -0.001 |
| VAGITNLK | 9 |  | 815.49779 | 54 | 0.001 |
| YVAGITNLK | 1 |  | 978.56163 | 53 | 0.000 |
| VPIGTLRPFL | 5 |  | 1112.68254 | 51 | 0.000 |
| (W)NVPIGTLRPFL(N) | 1 |  | 1226.72600 | 51 | 0.000 |
| VAGITNLKK | 3 |  | 943.59502 | 49 | 0.001 |
| LAVAVVR | 2 |  | 727.48247 | 49 | -0.000 |

Supplementary Table 2: Proteome Discoverer results of a nanoRP-LC-ESI-OT-MS^2^(HCD) measurement after digestion of BSA with trypsin. MASCOT search against unspecific *in-*silico digestion of mammalian taxonomy (UniProt-KB/SwissProt database).

| **Sequence** | **# PSM** | **Modifications** | **MH+ [Da]** | **IonScore** | **ΔM [Da]** |
| --- | --- | --- | --- | --- | --- |
| LGEYGFQNALIVR | 642 |  | 1479.79180 | 112 | -0.004 |
| KVPQVSTPTLVEVSR | 86 |  | 1639.93987 | 110 | 0.000 |
| KVPQVSTPTLVEVSR | 6 | Q4(Deamidated) | 1640.92436 | 110 | 0.003 |
| MPCTEDYLSLILNR | 6 | M1(Oxidation); C3(Carbamidomethyl) | 1740.82976 | 108 | 0.000 |
| VPQVSTPTLVEVSR | 2 | Q3(Deamidated) | 1512.82854 | 104 | 0.002 |
| MPCTEDYLSLILNR | 1 | C3(Carbamidomethyl) | 1724.83562 | 96 | 0.001 |
| TVMENFVAFVDK | 7 | | 1399.69560 | 93 | 0.003 |
| TVMENFVAFVDK | 14 | M3(Oxidation) | 1415.68877 | 93 | 0.002 |
| LGEYGFQNALIVR | 12 | N8(Deamidated) | 1480.78032 | 92 | 0.006 |
| VPQVSTPTLVEVSR | 9 |  | 1511.84307 | 90 | 0.000 |
| RHPEYAVSVLLR | 44 |  | 1439.81119 | 90 | -0.001 |
| DAFLGSFLYEYSR | 616 |  | 1567.74260 | 82 | 0.000 |
| YICDNQDTISSK | 3 | C3(Carbamidomethyl) | 1443.64311 | 80 | 0.001 |
| GLVLIAFSQYLQQCPFDEHVK | 9 | C14(Carbamidomethyl) | 2492.27080 | 77 | 0.006 |
| DAIPENLPPLTADFAEDK | 17 |  | 1955.96416 | 76 | 0.004 |
| LVNELTEFAK | 705 |  | 1163.63213 | 75 | 0.001 |
| DAIPENLPPLTADFAEDK | 2 | N6(Deamidated) | 1956.95293 | 75 | 0.009 |
| HLVDEPQNLIK | 652 |  | 1305.71573 | 73 | 0.000 |
| FYAPELLYYANK | 21 |  | 1491.75286 | 71 | 0.001 |
| PQVSTPTLVEVSR | 1 |  | 1412.77715 | 70 | 0.003 |
| RHPYFYAPELLYYANK | 5 |  | 2045.03081 | 69 | 0.003 |
| LKPDPNTLCDEFKADEK | 6 | C9(Carbamidomethyl) | 2019.96952 | 69 | 0.000 |
| LFTFHADICTLPDTEK | 3 | C9(Carbamidomethyl) | 1907.92160 | 68 | 0.001 |
| KQTALVELLK | 13 |  | 1142.71428 | 68 | 0.000 |
| AFLGSFLYEYSR | 3 |  | 1452.71758 | 68 | 0.002 |
| LGEYGFQNAL | 2 |  | 1111.54143 | 66 | 0.000 |
| FYAPELLYYANK | 1 | N11(Deamidated) | 1492.74236 | 66 | 0.006 |
| DAIPENLPPLTADFAEDKDVCK | 3 | C21(Carbamidomethyl) | 2458.18448 | 66 | 0.004 |
| SHCIAEVEK | 3 | C3(Carbamidomethyl) | 1072.51055 | 60 | 0.000 |
| LGSFLYEYSR | 2 |  | 1234.61003 | 60 | 0.002 |
| DDSPDLPK | 4 |  | 886.41557 | 60 | 0.000 |
| HPYFYAPELLYYANK | 6 |  | 1888.93045 | 58 | 0.004 |
| HPEYAVSVLLR | 9 |  | 1283.71208 | 57 | 0.001 |
| VSTPTLVEVSR | 2 |  | 1187.66374 | 56 | 0.001 |
| SQYLQQCPFDEHVK | 1 | C7(Carbamidomethyl) | 1778.82256 | 54 | 0.006 |
| KQTALVELLK | 2 | Q2(Deamidated) | 1143.69890 | 54 | 0.000 |
| DAIPENLPPLTADFAEDKD | 1 |  | 2070.98711 | 54 | 0.000 |
| RPCFSALTPDETYVPK | 3 | C3(Carbamidomethyl) | 1880.92327 | 53 | 0.005 |
| LKPDPNTLCDEFK | 1 | C9(Carbamidomethyl) | 1576.77104 | 53 | 0.003 |
| QTALVELLK | 2 | Q1(Deamidated) | 1015.60143 | 52 | -0.002 |
| HPYFYAPELLYYAN | 1 |  | 1760.83354 | 52 | 0.002 |
| FKDLGEEHFK | 30 |  | 1249.61954 | 52 | 0.002 |
| QTALVELLK | 1 |  | 1014.61962 | 51 | 0.000 |
| PNTLCDEFK | 1 | C5(Carbamidomethyl) | 1123.50969 | 51 | 0.001 |
| RHPEYAVSVL | 1 |  | 1170.62676 | 50 | 0.000 |
| SHKDDSPDLPK | 3 |  | 1238.60154 | 48 | 0.000 |
| LVNELTEFAK | 1 | N3(Deamidated) | 1164.61833 | 47 | 0.004 |
| SLHTLFGDELCK | 1 | C11(Carbamidomethyl) | 1419.69524 | 46 | 0.002 |
| HPYFYAPELLYY | 1 |  | 1575.75444 | 46 | 0.003 |
| AEFVEVTK | 6 |  | 922.48815 | 46 | 0.001 |
| KLVTDLTK | 2 |  | 917.56658 | 45 | 0.000 |
| LSQKFPK | 1 |  | 847.50133 | 42 | -0.002 |
| SEIAHR | 1 |  | 712.37370 | 41 | 0.000 |
| LVTDLTK | 13 |  | 789.47087 | 41 | 0.000 |
| YLYEIAR | 7 |  | 927.49590 | 40 | 0.001 |

Supplementary Table 3: Proteome Discoverer results of a nanoRP-LC-ESI-OT-MS^2^(HCD) measurement after digestion of hLTF with trypsin. MASCOT search against unspecific *in-*silico digestion of mammalian taxonomy (UniProt-KB/SwissProt database).

| **Sequence** | **# PSM** | **Modifications** | **MH+ [Da]** | **IonScore** | **ΔM [Da]** |
| --- | --- | --- | --- | --- | --- |
| ADAVTLDGGFIYEAGLAPYK | 1015 |  | 2071.03203 | 142 | -0.003 |
| AVTLDGGFIYEAGLAPYK | 14 |  | 1884.96941 | 118 | -0.005 |
| GEADAMSLDGGYVYTAGK | 48 | M6(Oxidation) | 1820.79863 | 117 | -0.002 |
| IDSGLYLGSGYFTAIQNLR | 102 |  | 2088.08208 | 116 | -0.002 |
| TLDGGFIYEAGLAPYK | 6 |  | 1714.86785 | 115 | -0.001 |
| DVTVLQNTDGNNNEAWAK | 10 |  | 1988.93132 | 111 | 0.000 |
| ESTVFEDLSDEAER | 30 |  | 1626.71208 | 105 | 0.001 |
| YLGPQYVAGITNLK | 1126 |  | 1536.84294 | 104 | 0.000 |
| CLAENAGDVAFVK | 2 | C1(Carbamidomethyl) | 1393.67656 | 100 | -0.002 |
| IDSGLYLGSGYFTAIQNLRK | 6 |  | 2216.16996 | 100 | -0.001 |
| ADAVTLDGGFIYEAGLAPY | 10 |  | 1942.94328 | 90 | -0.002 |
| LGSGYFTAIQNLR | 4 |  | 1439.76567 | 87 | 0.001 |
| KGGSFQLNELQGLK | 56 |  | 1518.82707 | 87 | 0.003 |
| VPPRIDSGLYLGSGYFTAIQNLR | 6 |  | 2537.34977 | 86 | -0.001 |
| LADFALLCLDGK | 6 | C8(Carbamidomethyl) | 1335.69707 | 85 | 0.000 |
| YLGPQYVAGITNLK | 4 | Q5(Deamidated) | 1537.82891 | 85 | 0.003 |
| SGLYLGSGYFTAIQNLR | 6 |  | 1859.96196 | 84 | -0.003 |
| SVNGKEDAIWNLLR | 10 | N3(Deamidated) | 1615.84221 | 83 | -0.002 |
| AMSLDGGYVYTAGK | 10 | M2(Oxidation) | 1448.67143 | 83 | -0.001 |
| SLDGGYVYTAGK | 12 |  | 1230.60100 | 82 | -0.001 |
| YLGPQYVAGITNLKK | 28 |  | 1664.93682 | 81 | 0.000 |
| KGGSFQLNELQGLK | 32 | Q6(Deamidated) | 1519.80997 | 81 | 0.000 |
| DSPIQCIQAIAENR | 6 | C6(Carbamidomethyl) | 1614.78862 | 77 | -0.002 |
| SVNGKEDAIWNLLR | 2 |  | 1614.86150 | 77 | 0.002 |
| GGSFQLNELQGLK | 32 |  | 1390.73210 | 77 | 0.001 |
| PVAAEVYGTER | 7 |  | 1191.60002 | 76 | -0.001 |
| RSDTSLTWNSVK | 8 |  | 1393.70696 | 75 | 0.000 |
| DGAGDVAFIR | 40 |  | 1020.51061 | 74 | 0.000 |
| GGFIYEAGLAPYK | 8 |  | 1385.71037 | 74 | 0.000 |
| GGSFQLNELQGLK | 21 | N7(Deamidated) | 1391.71709 | 72 | 0.000 |
| LRPVAAEVYGTER | 484 |  | 1460.78545 | 72 | 0.001 |
| THYYAVAVVKKG | 27 |  | 1335.74089 | 71 | -0.001 |
| IYEAGLAPYK | 4 |  | 1124.59795 | 70 | -0.001 |
| GAGDVAFIR | 4 |  | 905.48308 | 66 | -0.001 |
| SSQEPYFSYSGAFK | 12 |  | 1597.71636 | 65 | -0.001 |
| YYGYTGAFR | 117 |  | 1097.50408 | 64 | 0.000 |
| TGPPEPIEAAVAR | 12 |  | 1307.69414 | 64 | -0.001 |
| RPVEGYLAVAVVR | 6 |  | 1428.83183 | 64 | 0.000 |
| THYYAVAVVK | 76 |  | 1150.62493 | 63 | 0.000 |
| THYYAVAVVKK | 10 |  | 1278.71782 | 61 | -0.003 |
| SDTSLTWNSVK | 6 |  | 1237.60454 | 61 | -0.001 |
| LKQVLLHQQAK | 22 |  | 1305.79851 | 59 | -0.001 |
| VPSHAVVAR | 38 |  | 935.54033 | 57 | 0.000 |
| RPVAAEVYGTER | 2 |  | 1347.70329 | 57 | 0.002 |
| EDAIWNLLR | 14 |  | 1129.59892 | 56 | 0.000 |
| TAIQNLR | 4 |  | 815.47435 | 56 | 0.001 |
| KYLGPQYVAGITNLK | 12 |  | 1664.93680 | 56 | 0.000 |
| DGGYVYTAGK | 2 |  | 1030.48345 | 53 | -0.001 |
| QLNELQGLK | 2 |  | 1042.58916 | 52 | 0.000 |
| YVAGITNLK | 2 |  | 978.56017 | 52 | -0.002 |
| FQLFGSPSGQK | 307 |  | 1195.60991 | 52 | 0.000 |
| RKPVTEAR | 6 |  | 956.56316 | 51 | 0.000 |
| LRPVAAEVY | 5 |  | 1017.57176 | 50 | -0.001 |
| DSAIGFSR | 18 |  | 852.41979 | 50 | -0.001 |
| YLGPQYVAGITN | 6 |  | 1295.66326 | 49 | 0.000 |
| SFQLNELQGLK | 4 |  | 1276.68999 | 49 | 0.000 |
| PSHAVVAR | 11 |  | 836.47368 | 47 | 0.000 |
| MDKVER | 1 | M1(Oxidation) | 793.38671 | 47 | -0.001 |
| GGYVYTAGK | 2 |  | 915.45488 | 47 | -0.002 |
| FSYSGAFK | 3 |  | 906.43413 | 47 | -0.001 |
| THYYAVAVVKKGG | 9 |  | 1392.76201 | 46 | -0.001 |
| TAGWNVPIGTLR | 4 |  | 1284.70500 | 46 | -0.001 |
| SAIGFSR | 2 |  | 737.39324 | 46 | -0.001 |
| SPKFQLFGSPSGQKDLLFKD | 4 |  | 2239.17917 | 46 | 0.003 |
| FFSASCVPGADK | 2 | C6(Carbamidomethyl) | 1285.58806 | 46 | 0.000 |
| KSEEEVAAR | 6 |  | 1018.51573 | 44 | -0.001 |
| GEADAMSLDGGYVY | 5 | M6(Oxidation) | 1463.59929 | 44 | 0.000 |
| QVLLHQQAKFGRN | 5 |  | 1538.85440 | 44 | -0.001 |
| QVLLHQQAK | 36 |  | 1064.61968 | 40 | 0.000 |
| GPQYVAGITNLKK | 1 |  | 1388.78960 | 38 | 0.000 |
| YAVAVVK | 1 |  | 749.45525 | 35 | 0.000 |
